# The Relationship Between Central Auditory Tests and Neurocognitive Domains in Adults Living With HIV

**DOI:** 10.3389/fnins.2021.696513

**Published:** 2021-09-29

**Authors:** Christopher E. Niemczak, Jonathan D. Lichtenstein, Albert Magohe, Jennifer T. Amato, Abigail M. Fellows, Jiang Gui, Michael Huang, Catherine C. Rieke, Enica R. Massawe, Michael J. Boivin, Ndeserua Moshi, Jay C. Buckey

**Affiliations:** ^1^Space Medicine Innovations Laboratory, Geisel School of Medicine, Dartmouth College, Hanover, NH, United States; ^2^Dartmouth-Hitchcock Medical Center, Lebanon, NH, United States; ^3^Department of Psychiatry, Geisel School of Medicine, Dartmouth College, Hanover, NH, United States; ^4^Department of Otorhinolaryngology, Muhimibili University of Health and Allied Sciences, Dar es Salaam, Tanzania; ^5^Department of Data Science, Geisel School of Medicine, Dartmouth College, Hanover, NH, United States; ^6^Department of Psychiatry, Michigan State University, East Lansing, MI, United States

**Keywords:** HIV, cognition, central auditory processing, attention, auditory disease, cognitive processing speed, Africa South of the Sahara

## Abstract

**Objective:** Tests requiring central auditory processing, such as speech perception-in-noise, are simple, time efficient, and correlate with cognitive processing. These tests may be useful for tracking brain function. Doing this effectively requires information on which tests correlate with overall cognitive function and specific cognitive domains. This study evaluated the relationship between selected central auditory focused tests and cognitive domains in a cohort of normal hearing adults living with HIV and HIV– controls. The long-term aim is determining the relationships between auditory processing and neurocognitive domains and applying this to analyzing cognitive function in HIV and other neurocognitive disorders longitudinally.

**Method:** Subjects were recruited from an ongoing study in Dar es Salaam, Tanzania. Central auditory measures included the Gap Detection Test (Gap), Hearing in Noise Test (HINT), and Triple Digit Test (TDT). Cognitive measures included variables from the Test of Variables of Attention (TOVA), Cogstate neurocognitive battery, and Kiswahili Montreal Cognitive Assessment (MoCA). The measures represented three cognitive domains: processing speed, learning, and working memory. Bootstrap resampling was used to calculate the mean and standard deviation of the proportion of variance explained by the individual central auditory tests for each cognitive measure. The association of cognitive measures with central auditory variables taking HIV status and age into account was determined using regression models.

**Results:** Hearing in Noise Tests and TDT were significantly associated with Cogstate learning and working memory tests. Gap was not significantly associated with any cognitive measure with age in the model. TDT explained the largest mean proportion of variance and had the strongest relationship to the MoCA and Cogstate tasks. With age in the model, HIV status did not affect the relationship between central auditory tests and cognitive measures. Age was strongly associated with multiple cognitive tests.

**Conclusion:** Central auditory tests were associated with measures of learning and working memory. Compared to the other central auditory tests, TDT was most strongly related to cognitive function. These findings expand on the association between auditory processing and cognitive domains seen in other studies and support evaluating these tests for tracking brain health in HIV and other neurocognitive disorders.

## Introduction

Central auditory function, reflected in tests of speech perception in background noise, correlates with cognition (Watson, 1991; Hallgren et al., 2001; Anderson and Kraus, 2010; Hoover et al., 2017; Panza et al., 2018; Danielsson et al., 2019; Humes, 2020, 2021), including cognitive dysfunction due to mild cognitive impairment (MCI), Alzheimer’s disease (Gates et al., 1996; Idrizbegovic et al., 2011), and HIV (Zhan et al., 2017b; Buckey et al., 2019; White-Schwoch et al., 2020; Niemczak et al., 2021). This suggests central auditory tests might be useful for tracking cognitive dysfunction in populations with disordered neuro-cognitive processing. Yet, the relationship between central auditory function and cognition is multifactorial and questions remain regarding the correlation of central auditory tests with specific cognitive domains (i.e., processing speed, working memory, etc.). The ability to perceive, understand, and respond to a conversational partner in background noise encompasses a variety of neurocognitive domains (Gates et al., 1996; Anderson et al., 2013). While conversing in a noisy environment may seem like a simple, common task, detecting quick acoustic changes in an auditory stream and understanding the content, requires fast and accurate processing in the auditory processing pathway–including higher-level cognitive processing. The correlation between central auditory function and cognition suggests that auditory information is not only processed by the cochlea and auditory pathway, but also by other associative cortical areas (Harris et al., 2012; Palmer and Musiek, 2014; Sardone et al., 2019; Song et al., 2020). Our previous work has shown a relationship between central auditory test performance and cognitive performance, which suggested that central auditory tests might be useful for tracking cognitive performance in people living with HIV (PLWH) (Maro et al., 2014; Zhan et al., 2018; Buckey et al., 2019; Niemczak et al., 2021). The goal of this study was to evaluate the relationship between central auditory tests and neurocognitive domains in adults living with HIV and HIV-negative (HIV–) controls. Specifically, we examined how three central auditory tests relate to the specific cognitive domains of processing speed, learning, and working memory. The aim was to provide focused results on specific relationships of auditory processing and distinct neurocognitive domains to inform multifactorial longitudinal analyses in HIV and other neurocognitive disorders.

The worldwide prevalence of HIV/AIDS is approximately 37.9 million, with sub-Saharan African countries accounting for two thirds of the global HIV burden (Prevention, February 5, Centers for Disease Control and Prevention, 2020). Advances in understanding HIV replication, tracking immunologic progression, and using combination antiretroviral therapy (cART), have resulted in reduced viral loads and a drastic reduction in HIV mortality (Saylor et al., 2016). While rates of asymptomatic and mild neurocognitive dysfunction remain stable, cART has resulted in a significant decline in HIV-related dementia (Saylor et al., 2016). Despite this reduction in the severest forms of cognitive impairment, neurocognitive dysfunction persists in a subset of PLWH, even among those consistently taking cART and those with suppressed viral loads (Heaton et al., 2015). Identifying, tracking, and potentially predicting the development of neurocognitive dysfunction in this population would provide crucial benefits for PLWH. Identifying biomarkers for brain health in PLWH is important for reducing mortality, morbidity, and disease progression (Saylor et al., 2016). Cognitive impairment can lower treatment adherence and quality of life (Ettenhofer et al., 2010). Variability in how neurocognitive dysfunction presents in HIV relates to the diffuse nature of the disease’s impact upon the central nervous system (Zhan et al., 2017a). Per recommendations from the National Institute of Neurological Diseases and Stroke, comprehensive neuropsychological assessment is considered the gold standard in assessing and monitoring neurocognition in PLWH (Antinori et al., 2007). This form of assessment covers a variety of cognitive domains, both broad and specific, providing an understanding of global function and particular domain-based skills (e.g., language, attention, memory). Neuropsychological assessment, however, is costly, time consuming, and requires specialized training for interpretation. It is also sensitive to education and cultural background. Access to such evaluations in resource limited settings, where the burden of HIV and other neurocognitive diseases tends to be significantly higher than in developed countries, is not always feasible. Additionally, finding culturally and linguistically appropriately normed measures is challenging (Robertson et al., 2009). Central auditory focused tests offer an attractive option in these settings because the tests are easy to explain, unlikely to be sensitive to education or cultural background, and can be repeated over time.

People living with HIV have differences in brain regions and functions necessary for auditory processing including gray matter atrophy, axonal injury, loss of axonal density, and diffuse white matter abnormalities in the internal capsule, thalamus, and corpus collosum (Zhan et al., 2017a; Kuhn et al., 2018). The auditory system provides a useful tool for assessing brain function because processing auditory information is neurologically demanding. Most people are familiar with tests of peripheral hearing (e.g., a threshold audiogram), but centrally focused auditory tests involve much more than just assessing the quietest tone an individual can detect. After the cochlea converts sound waves into nerve signals the brain must quickly perform a series of complex functions to determine the meaning of the content. Speech perception, particularly interpreting speech in noise, engages several cortical and subcortical centers (Kotz and Schwartze, 2010; Specht, 2014). This involves neural pathways throughout the brainstem and into the cortex that integrate with high-level linguistic and cognitive systems, such as processing speed and working memory (Rudner and Lunner, 2014; Pichora-Fuller et al., 2016). Previous studies have shown that more complex auditory tasks relate to cognition beyond peripheral hearing sensitivity (Danielsson et al., 2019). A recent study by Danielsson et al. found associations between auditory processing tasks of temporal-order identification and gap detection with semantic long-term memory and working memory. Auditory thresholds had no significant effect on any of the cognitive measures. What makes central auditory focused tests so appealing is that they are relatively short (a gap detection test takes 5 min), easy to explain (the hearing-in-noise test and triple digit task involve identifying words or numbers in background noise), can be repeated, and do not require trained administrators. This is particularly important for deploying these tests in the developing world where normative cognitive data often do not exist, and education levels are variable. These measures would be a major advance for following PLWH in the developing world. Further understanding the relationship of central auditory tests with specific neurocognitive domains could also provide detailed knowledge for targeted longitudinal analyses to better assess, track, and potentially predict neurodegeneration in those with HIV and other neurocognitive diseases.

Our group has shown that PLWH with normal peripheral hearing are more likely to report trouble understanding speech in noise compared to controls (Maro et al., 2014; Zhan et al., 2017b). These studies suggest worse performance on measures of speech-in-noise identification may reflect damage to the central nervous system resulting in cognitive impairment (Buckey et al., 2019). Previous studies have shown a relationship between cognition and auditory processing ability such as speech perception in noise (Pichora-Fuller et al., 1995; Wong et al., 2009; Zekveld et al., 2011), auditory temporal ordering (Szymaszek et al., 2009; Danielsson et al., 2019; Humes, 2020;2021), and gap detection testing (Harris et al., 2010). In our previous work, we have shown a significant relationship between the ability to understand speech in noise and cognitive status in PLWH (Zhan et al., 2017b). Also, using fMRI we have shown activation in frontal areas during a challenging speech-in-noise task (Song et al., 2020) showing that challenging speech-in-noise tasks involve areas beyond the auditory cortex. In addition, a growing body of literature suggests central auditory processing deficits in PLWH on self-report, neurophysiological, and audiological measures (White-Schwoch et al., 2020; Niemczak et al., 2021). The relationship between measures of auditory processing and cognition likely relates to the similarities in specific neurocognitive domains used for advanced processes of auditory perception and higher-order cognition (Danielsson et al., 2019). The goal of this study was to examine a selection of central auditory tests and their relationship to specific cognitive domains to help select and inform which tests might be most sensitive in a longitudinal study to help detect cognitive changes over time. To do this, we focused on tests we believed had a strong central auditory component. We deliberately included individuals who had normal peripheral auditory function (i.e., normal audiograms) and selected tests that involve temporal processing and speech perception in noise. We hypothesized that central auditory tests would be associated with cognitive function in three domains of processing speed, learning, and working memory in PLWH. By using cognitive measures with various neurocognitive domains (speed of processing, learning, working memory), this analysis would also examine the specific domains of cognition that most strongly relate to central auditory performance. If central auditory tests are related to certain aspects of cognitive function, these measures might also provide a quantitative, time efficient, and repeatable measure of cognitive function in the developing world. In addition, once these relationships are identified, longitudinal analyses could be used to develop a predictive auditory screening tool related to multiple domains of cognitive function.

## Materials and Methods

### Study Design and Participants

This prospective cohort study design was derived from a longitudinal study of HIV-infected adults (all taking cART) in Dar es Salaam, Tanzania. At the time of data collection for this analysis, we had gathered auditory and cognitive information from 259 HIV+ (mean 40.9 years) to 76 HIV– (mean 32.3 years) individuals from the larger study database. See Table 1 for a complete review of sample demographics.

**TABLE 1:** Summary of study demographics between HIV groups.

		HIV+(*n* = 259)	HIV–(*n* = 76)	*P-*value
**Age (mean, SD)**		40.9 (12.0)	32.3 (10.6)	<0.001
**Gender (n, %)**	*Male*	74 (29%)	31 (40%)	-
	*Female*	185 (71%)	39 (60%)	-
**Years of education (mean, SD)**		8.92 (2.61)	10.3 (2.71)	<0.001
**Pure tone average (dB HL mean, SD)**	*Right*	7.33 (4.86)	4.82 (5.50)	0.001
	*Left*	6.48 (5.09)	4.74 (5.49)	0.002
**CD4 counts (mean, SD)**		681.6 (318.2)	787.1 (212.1)	<0.001
**Lowest CD4 counts (mean, SD)**		406.7 (205.8)	659.9 (210.0)	<0.001

*Mean and standard deviation (SD) of each demographic variable for HIV groups. *P*-values were calculated using the Mann-Whitney *U*-tests due to differences in distribution of groups, which were found to be significant across all tests (all ≤0.002).*

### Procedures

The institutional review boards of both Dartmouth College and the Muhimbili University of Health and Allied Sciences approved this study’s research protocol. All research was completed in accordance with the Helsinki Declaration. All participants were obligated to provide written informed consent. Participants consisted of PLWH and HIV– adults who were tested at the Infectious Disease Center in Dar es Salaam, Tanzania. To ensure accuracy of analysis and control for variables that could affect central auditory and cognitive function, we used a series of data selection techniques. Individuals were excluded if they had abnormal hearing sensitivity (>25 dB HL from 0.5 to 4 kHz) or abnormal middle ear function. Individuals were also excluded if they had a positive history of ear drainage, concussion, significant noise or chemical exposure, neurological disease, mental illness, ototoxic antibiotics (e.g., gentamycin), or chemotherapy. This selection technique resulted in 385 individuals, but only 335 individuals with complete central auditory variables (i.e., no missing auditory data) were selected for the study. We wanted every subject to have completed all the central auditory focused tests. The demographics of this sample population are provided in Table 1. To reduce the variation from using a single measurement from a single experimental session (cross-sectional sample), we used the mean from multiple visits over time. To do this, we plotted each subject’s cognitive and central auditory tests over time and identified outliers by plotting a regression line to the data and removing values that were >2 standardized residuals from the regression line. After removing outliers, we took the average of each subject’s test scores over time to create one data point for each subject.

Audiological testing was performed using a hearing assessment system built by the Creare, LLC. Creare’s wireless noise attenuating headset (CWNAH) has the device speakers mounted in highly noise attenuating ear cups. The attenuation provided by this headset is better than any currently available commercial hearing test device as measured by an independent laboratory according to the relevant ANSI standards (Meinke et al., 2017). Before starting the audiological evaluation, patients had an otoscopic exam and cerumen was removed as needed. All subjects completed a health history questionnaire that asked about health conditions that might affect their hearing or central nervous system (e.g., head trauma, other central nervous system infection). To verify normal middle ear status, tympanometry at 226 Hz was performed on both ears using a Madsen Otoflex 100 (GN Otometrics, Denmark). Individuals with abnormal tympanograms (Type B or C) were referred for treatment and subsequent reevaluation.

Hearing thresholds were measured at frequencies 500, 1000, 2000, and 4000 Hz using a Békésy-like tracking procedure as described previously (Maro et al., 2014). Thresholds of 25 dB HL or better for each ear across the aforementioned frequencies were considered normal. Pure tone averages (PTA) were calculated by taking the mean of all measured frequencies. Measures of central auditory processing included gap detection, speech-in-noise, and digits-in-noise testing. The Kiswahili language version of the Hearing in Noise Test (HINT) and the Kiswahili Triple Digit Test (TDT) were used to assess speech perception in noise. The HINT was administered in three test conditions: Noise Front, Noise Right, and Noise Left. In each HINT test, a different list of 20 sentences was presented in random order in the presence of the masking noise spectrally matched to the long-term average of the target material. The presentation level of the noise remained fixed at 65 dB (A-weighting), and the test instrument adjusted the level of each sentence adaptively depending on whether the test administrator indicated that the previous sentence was repeated correctly. The presentation level of the sentence was reduced if the previous sentence was repeated correctly and increased if the previous sentence was repeated incorrectly. This adaptive procedure was used to determine the presentation level of each sentence in the list. The average presentation level of all sentences after the first four sentences defined the speech reception threshold (SRT) for the test condition expressed as a signal to noise ratio (SNR). A composite SNR of all three noise conditions was calculated and used as the primary variable of interest for the HINT.

In the TDT, recordings of natural productions of three-digit triplets such as 3-5-9 (spoken as “tatu-tano-tisa” in Kiswahili) were used as target stimuli (Kiswahili numbers below 10 have the same number of syllables). All digit triplets were produced and recorded by a male speaker in a soundproof booth. Digit triplet recognition was tested in the presence of competing Schroeder-phase masking noise. The test included 30 total presentations of pseudorandom digit triplets with 6 practice presentations. Presentations were presented in pairs of positive and negative phase maskers. Each pair was presented at the same SNR. The ordering of the masker was randomized for each pair. The test started at a 0-dB initial SNR with the masker fixed at 75 dB SPL. SNRs were then adjusted after each presentation or pair of presentations by varying the target level. 2.0 dB SPL was added to the target level for each incorrect digit and 2.0 dB SPL was subtracted for each correct digit from the previous positive-phase presentation. A speech reception threshold was calculated as the SNR of the last 7 positive-phase presentations, which was used as the primary variable of interest. Completing one list of 30-digit triplets took 3-6 min.

The Gap Detection Test (GAP) determines a participant’s ability to identify short gaps in noise by pushing a button when they first identify a break in noise. The details of the gap detection test have been published previously (Maro et al., 2014). We implemented an adaptive gap detection algorithm using a single staircase. Gaps occur randomly in the middle portion of 4.5 s of white noise delivered at 65 dB SPL. No gaps were presented in the first or last second. If the subject misidentified 2 gaps, in a row or 3 gaps overall, the staircase “reversed,” and the gap length increased. In this way, the staircase algorithm converged to the subject’s gap threshold. The test started at a gap length of 20 m sec and continued until the subject completed 10 reversals or a total of 120 presentations. From these gap tests a plot of the percentage of time a gap was correctly detected vs. gap length was produced. This curve can be fit using the Hill equation to calculate the gap length where 50% of the gaps were detected correctly (Grigoryan et al., 2013). These values were used in the analysis. The subjects received training in the gap test with both a training video and a screen that provided both auditory and visual feedback. The operator presented gaps to the subject until the subject comprehended the task.

The Kiswahili version of the Montreal Cognitive Assessment (MoCA) was used as a screening measure of cognitive function. The MoCA has been used in diverse populations and has demonstrated reliability and validity with the Tanzanian population (Vissoci et al., 2019). This measure assesses short-term and long-term memory recall, visual spatial abilities, executive functioning, attention, concentration, and working memory. The maximum score achievable is 30 points and a correction was implemented based on years of education.

Computerized neurocognitive assessments were conducted using the Cogstate and Test of Variables of Attention (TOVA). All cognitive assessments utilized visual stimuli, not auditory stimuli, to test neurocognitive function (apart from the instructions). The Cogstate^1^ is comprised of multiple tests assessing a variety of domains. The Cogstate battery, was chosen because it uses culturally neutral stimuli (e.g., playing cards) to ensure that the assessment is not limited by a participant’s level of education. Card games are popular in Tanzania, so the playing card approach was familiar to the cohort. The Cogstate tasks are computer-based and designed for repeated administration. The Cogstate battery has been used to assess cognitive function in patients with HIV and has been shown to correlate well with standard neuropsychological test batteries (Cysique et al., 2006; Overton et al., 2011; Bloch et al., 2016; Kamminga et al., 2017). In addition, the Cogstate includes tasks known to be sensitive to cognitive domains affected in adults (Hammers et al., 2011, 2012; Bloch et al., 2016; Boivin et al., 2016). We chose outcomes measures of working memory and learning from the Cogstate to include in our analysis. The Test of Variables of Attention (TOVA. Version 8.0) was also administered to all subjects as it has been used with sub-Saharan African populations across a wide range of studies (Bangirana et al., 2015; Boivin et al., 2019). The TOVA has several advantages because it uses visual stimuli, measures response times precisely (±1 m sec), is language- and culture-free, and has a history of use in resource-challenged areas (Ruel et al., 2012). Table 2 has a list of the measures, their cognitive domains, and the outcome variables used in our analyses.

**TABLE 2:** Descriptions of cognitive measures.

Cognitive measure	Domain assessed	Test description	Outcome measure
***MoCA***			
Kiswahili MoCA	Neurocognitive Screening	Screens short- and long-term memory recall, visual spatial abilities, executive functioning, attention, concentration, and working memory.	A subject is able to obtain a total of 30 points. Higher scores represent better performance.
***TOVA***			
Response Time *(RT)*	Attention, processing speed *(RT-ms)*	Correct response time mean. Measures quickness of response time in milliseconds.	The mean response time of the correct responses. Lower = better.
Ex-Gaussian μ *(ExG*μ*)*	Attention, processing speed *(ExG*μ*-ms)*	The mean response time (in milliseconds) of the correct responses, modeled using the Ex-Gaussian distribution.	This score takes into account the skew in distribution of reaction time scores. Lower = better.
***Cogstate***			
One Card Learning *(OCL)*	Visual leaning accuracy* *(OCL-acc)*	Accuracy of performance; arcsine square root proportion correct.	Accuracy is the primary outcome for OCL. Higher scores = better.
	Visual learning speed *(OCL-lmn)*	Speed of performance; mean of the log_10_ transformed reaction times for correct responses.	Speed of processing is the secondary outcome measure for OCL. Lower score = better
One Back Test *(ONB)*	Working memory accuracy *(ONB-acc)*	Accuracy of performance; arcsine square root proportion correct.	Accuracy is the secondary outcome for ONB. Higher scores = better.
	Working memory speed* (ONB-lmn)	Speed of performance; transformed reaction times for correct responses.	Speed of processing is the primary measure for ONB. Lower score = better

*All cognitive measures, the domain they assess, a description of the test, and the outcome measures are listed for the entire study. MoCA, Montreal Cognitive Assessment; TOVA, Test of Variables of Attention. ^∗^Indicates the primary variable of interest for Cogstate subtest.*

### Statistical Analysis

Data were analyzed and plotted using MATLAB^®^ 2020b. As stated above, a set of cognitive outcome variables were determined *a priori* based on the sensitivity of specific domains, their correlation to standard neuropsychological test batteries, and cognitive domains likely to be affected by HIV (see Table 2). We first tested simple group mean differences using non-parametric procedure (Mann-Whitney U-test). A bootstrapping method was used to examine the relationship of central auditory tests to cognitive measures. This resampling technique was used to estimate the mean and standard deviation of the proportion of variance (R^2^) and overall significance (*p*-Value) of the linear relationship between each central auditory test and cognitive measure. We randomly sampled ∼60% of the cohort (200 individuals) 5000 times for each central auditory/cognitive relationship. To examine the effects of age and HIV, we used general linear models to assess the association between central auditory tests and cognitive measures with age and HIV included in the model [model specification - (*Cognitive measure* ∼ *Gap* + *HINT* + *TDT* + *HIV status* + *Age*)]. Previous studies have shown a strong relationship between age, auditory tests, and cognitive measures (see Humes et al., 2012 for review). This analysis method resulted in 7 models, one for each cognitive measure. To adjust for multiple comparisons, we used an α level of <0.005. For all model analyses, a variance inflation factor was calculated to assess collinearity among the predictor variables (Gap, HINT, and TDT). All variance inflation factors were <1.8 indicating low levels of collinearity between predictor variables. This provided an alternative way to examine the sampling distribution of proportion of variance and overall significance.

## Results

### Overall Mean HIV+ and HIV– Group Comparisons

Overall demographic differences between groups showed that HIV + individuals were older, less educated, and had pure tone averages that were higher than the HIV– group. See Table 2 for demographic differences. Mean differences between central auditory and cognitive measures also revealed significant HIV group differences. See Table 3 for mean and standard deviations of cognitive and central auditory processing outcome variables. All central auditory variables revealed highly significant differences between HIV groups (*p* ≤ 0.012). MoCA, TOVA, and Cogstate also revealed significant differences between groups (*p* ≤ 0.037). Importantly, these differences do not show the association between auditory and cognitive variables.

**TABLE 3:** Mean central auditory and cognitive performance differences between HIV groups.

		Mean performance scores		
Measure	Subtest	HIV + (SD)	HIV– (SD)	*P*-value
Central auditory processing	Gap Detection Test (*GAP* - ms)	3.65 (1.37)	3.00 (1.18)	<0.001
	Hearing in Noise Test (*HINT* - dB SNR)	–10.7 (0.99)	–11.1 (1.01)	0.012
	Triple Digit Test (*TDT* - dB SNR)	–18.1 (4.70)	–20.1 (4.63)	<0.001
MoCA	Total Score (# correct)	26.6 (2.86)	27.5 (2.30)	0.012
TOVA	Reaction Time *(RT* - *ms)*	395.1 (62.9)	364.7 (63.2)	<0.001
	Ex-Gaussian μ *(ExG*μ - *ms)*	324.7 (59.0)	296.1 (51.5)	<0.001
Cogstate	*One Card Learning – accuracy *(OCL*-*acc)*	0.94 (0.10)	0.96 (0.10)	0.013
	One Card Learning – speed of processing *(OCL*-*lmn)*	3.17 (0.11)	3.10 (0.09)	<0.001
	*One Back Test – speed of processing *(ONB*-*lmn)*	3.08 (0.10)	3.01 (0.09)	<0.001
	One Back Test – accuracy *(ONB*-*acc)*	1.07 (0.20)	1.12 (0.17)	0.037

*Mean and standard deviation (SD) are displayed for HIV + and HIV– groups.*

Significant differences were found on all central auditory variables and cognitive measures. *P*-values were calculated using Mann-Whitney *U*-tests (non-parametric) due to differences in distribution between HIV groups. *Indicates the primary outcome measures for Cogstate subtest. Each measure is displayed in raw score units along with the applicable abbreviation in italic.

### Relationship of Specific Central Auditory Tests to Cognitive Measures

To evaluate the relationship of central auditory variables to specific cognitive domains, we used a bootstrapping method to assess the relationship between each individual auditory variable and each cognitive measure. The most significant results were the relationships of the TDT to the MoCA, OCL-lmn, and ONB-lmn (all mean *p* < 0.001) with mean R^2^ values of 0.142, 0.113, and 0.120, respectively (Table 4). All central auditory focused tests were significantly related to the MoCA, but the TDT relationship was much stronger than for either the HINT or Gap and was the only significant measure when multiple comparisons were considered. The Gap test was significantly related to the TOVA measures, but neither the HINT nor TDT were related to the TOVA. The strongest results were for the TDT with the OCL and ONB speed of processing measures. The HINT was only weakly related to these measures. Interestingly, the HINT was significantly related to the OCL and ONB accuracy measures, while the TDT and Gap were not.

**TABLE 4:** Bootstrap results.

			Bootstrap results			
Cognitive variable	Response variable	Predictor variable	Mean R^2^	Std. R^2^	Mean *p*-Value	Std. *p*-Value
*MoCA*	*Total score*	Gap	0.036	0.016	0.033	0.056
		HINT	0.042	0.022	0.034	0.072
		TDT	0.142	0.028	<0.001	<0.001
*TOVA*	*Response time*	Gap	0.039	0.017	0.021	0.043
		HINT	0.012	0.004	0.498	0.205
		TDT	0.019	0.010	0.158	0.169
	*ExG*μ	Gap	0.030	0.016	0.045	0.076
		HINT	0.002	0.004	0.582	0.260
		TDT	0.011	0.008	0.234	0.196
*Cogstate*	OCL-acc*	Gap	0.011	0.009	0.271	0.023
		HINT	0.037	0.017	0.035	0.073
		TDT	0.018	0.010	0.132	0.144
	*OCL-lmn*	Gap	0.029	0.017	0.056	0.089
		HINT	0.038	0.018	0.024	0.048
		TDT	0.113	0.025	<0.001	0.001
	*ONB-acc*	Gap	0.011	0.009	0.254	0.232
		HINT	0.096	0.018	0.012	0.032
		TDT	0.032	0.017	0.045	0.076
	ONB-lmn*	Gap	0.027	0.014	0.061	0.093
		HINT	0.032	0.017	0.044	0.079
		TDT	0.120	0.022	<0.001	<0.001

The results of resampling between individual central auditory and cognitive measures are displayed below. 200 random samples of the 335 subject cohort were chosen over 5000 iterations to calculate the mean and standard deviation (Std.) R^2^ and *p*-Value. The relationship of TDT and MoCA, OCL-lmn, and ONB-lmn showed the largest R^2^ with mean *p*-Values below 0.001. *Indicates the primary outcome measures for Cogstate subtest. The colored values indicate significant of *p* < 0.005.

### Association of Cognitive Measures to Central Auditory Measures Including Age and HIV Status

**FIGURE 1:**
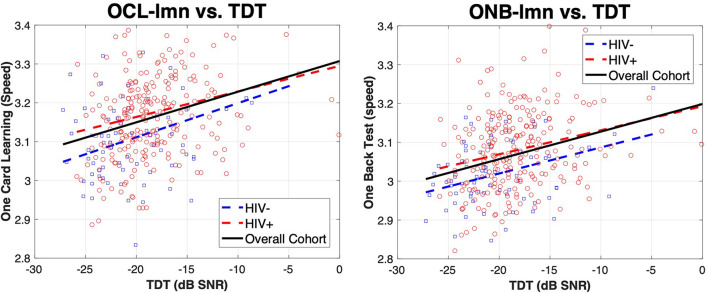
Relationship of OCL-lmn and ONB-lmn with TDT for HIV– and HIV + individuals. Panels show the relationship of OCL-lmn (left) and ONB-lmn (right) for HIV– (blue squares) and HI + (red circles). Lines indicate a least-squares fit for HIV– (blue dotted line), HIV + (red dotted line), and the overall cohort (black solid line). OCL-lmn is the mean of the log_10_ transformed reaction times for correct responses during the One Card Learning task. This task was to quickly and accurately recognize if a playing card has been presented before throughout the duration test. Lower scores equate to better performance. ONB-lmn is also the log_10_ transformed reaction times for the One Back Test using playing cards. Results show significant positive relationships between TDT and OCL-lmn and ONB-lmn.

We evaluated the association of cognitive test domains to central auditory tests, including age and HIV status in the model. Overall, we found significant associations between multiple cognitive and central auditory test variables. Table 5 shows the results of all linear regression models. MoCA scores were significantly associated with the TDT (*p* < 0.001), but not with any other variable. TOVA response time (RT) and ex-gaussian μ (ExGμ) were significantly associated with age (*p* < 0.001) but no central auditory/cognitive relationship was found to be significant. Cogstate OCL accuracy (OCL-acc) was only significantly associated with HINT (*p* = 0.004), while the speed of learning (OCL-lmn) was significantly associated with age (*p* < 0.001) and TDT (*p* < 0.001). Cogstate ONB accuracy (ONB-acc) was significantly associated with HINT (*p* = 0.003), while working memory speed (ONB-lmn) was significantly associated with age (*p* = 0.001) and TDT (*p* = 0.002). Together, these results suggest that: (1) Gap was not significantly related to any of the cognitive measures independent of age; (2) The HINT was significantly related to OCL-acc and ONB-acc, and (3) The TDT showed the strongest associations with significant relationships to the MoCA, OCL-lmn, and ONB-lmn. Age also showed strong associations with TOVA response time, ExGμ, OCL-lmn, and ONB-lmn. R^2^ values were also strongest for the OCL-lmn (0.253) and ONB-lmn (0.257) models. Surprisingly, HIV status was not significant in any model and age was not significantly related to the MoCA. Figure 1. shows plots of OCL-lmn and ONB-lmn for the TDT. The graphs show a significant positive trend for the entire cohort and for the HIV + group to perform slightly worse on both measures.

**TABLE 5:** Linear regression results.

				Linear regression results			
Cognitive variable	Response variable	Predictor variable	Beta	Std. Err.	t-Stat	*p*-Value	R^2^
*MoCA*	*Total score*	HIV	0.060	0.387	0.156	0.876	0.177
		Age	–0.025	0.014	–1.769	0.078	
		TDT	–0.173	0.036	–4.780	<0.001	
		HINT	–0.310	0.151	–2.052	0.041	
		Gap	–0.174	0.118	–1.478	0.140	
*TOVA*	*Response time*	HIV	–11.168	8.771	–1.273	0.204	0.145
		Age	1.629	0.323	5.049	<0.001	
		TDT	–0.171	0.814	–0.210	0.834	
		HINT	–3.052	3.513	–0.869	0.386	
		Gap	3.239	2.729	1.187	0.236	
	*ExG*μ	HIV	–9.722	7.741	–1.256	0.210	0.198
		Age	1.982	0.284	6.984	<0.001	
		TDT	–0.661	0.718	–0.921	0.358	
		HINT	–2.589	3.073	–0.842	0.400	
		Gap	0.466	2.397	0.195	0.084	
*Cogstate*	OCL-acc*	HIV	0.008	0.014	0.579	0.563	0.069
		Age	0.000	0.001	–0.853	0.394	
		TDT	–0.001	0.001	–0.634	0.526	
		HINT	–0.016	0.006	–2.792	0.004	
		Gap	–0.004	0.004	–0.806	0.421	
	*OCL-lmn*	HIV	–0.015	0.015	–0.976	0.330	0.253
		Age	0.003	0.001	5.813	<0.001	
		TDT	0.005	0.001	3.358	0.001	
		HINT	0.011	0.006	1.757	0.080	
		Gap	–0.001	0.005	–0.265	0.791	
	*ONB-acc*	HIV	0.005	0.026	0.187	0.852	0.097
		Age	–0.002	0.001	–2.132	0.034	
		TDT	–0.003	0.002	–1.258	0.209	
		HINT	–0.030	0.010	–2.870	0.003	
		Gap	–0.003	0.008	–0.341	0.733	
	ONB-lmn*	HIV	–0.019	0.013	–1.489	0.137	0.257
		Age	0.003	0.000	6.076	0.001	
		TDT	0.004	0.001	3.109	0.002	
		HINT	0.006	0.005	1.255	0.211	
		Gap	–0.002	0.004	–0.472	0.637	

Results display the association between central auditory and cognitive measures as well as HIV status and age. Models show decreased performance on central auditory tests are significantly associated with degraded cognitive measures (Model specification: *Cognitive measure* ∼ *HIV status* + *Age* + *TDT* + *HINT* + *Gap*). Significant values are highlighted in gray. *Indicates the primary outcome measures for Cogstate subtest. T-stat was calculated by dividing the Beta (coefficient) by the standard error.

## Discussion

The findings of the current study support and build upon previous work in understanding how central auditory measures and cognition are associated. The results showed that central auditory tests were significantly associated with cognitive abilities across areas of learning and working memory on the Cogstate, but not response time as measured by the TOVA. The HINT and TDT showed the strongest associations with learning and working memory on the Cogstate. Specifically, the TDT showed the strongest relationship to OCL-lmn and ONB-lmn in the linear models and with the bootstrapping resampling method. Surprisingly, HIV status was not significantly associated with any cognitive measure. These findings support the idea that central auditory tests assess cognitive functions related to the domains of learning and working memory. Longitudinal results are needed to determine whether central auditory focused tests could be used to track cognitive function over time in patients with particular conditions that affect the central nervous system, such as HIV and other neurocognitive diseases.

With speech-in-noise measures, we found that subjects who performed better on HINT and TDT also performed better on OCL and ONB tasks related to learning and working memory. Speech perception in noise has been linked to multiple cognitive functions (i.e., executive and attentive functions) (Anderson and Kraus, 2010; Anderson et al., 2013; Moore et al., 2014). The rationale for this link is based on the complex acoustic processing necessary to perceive word representations and phonemes accurately, extract the meaning of the message within background noise successfully, and remember the message from beginning to end to respond correctly. This process requires cognitive-linguistic abilities, including working memory to attend to and recall what is being said (Craik, 2007; Anderson and Kraus, 2010; Rönnberg et al., 2013). Success in the learning domain, as evidenced by OCL tasks, also requires recruitment of complex neural networks across a broad range of cortical and subcortical areas (Koziol and Budding, 2009; Du Plessis et al., 2017). Recently, using fMRI we found increased activation in the right superior and middle frontal gyrus during speech perception in noise in a cohort of normal hearing Mandarin listeners (Song et al., 2020). These two frontal sub-regions are relevant for tone perception, phonological working memory, and orientation of attention (Husain et al., 2006; Japee et al., 2015). Therefore, this provides evidence that speech-in-noise tests are tapping into areas of the brain also associated with tests such as the ONB and OCL.

The strongest relationships were seen with the TDT. The TDT is a closed set task (numbers 1-9) with no spatial adjustment and uses Schroder-phase masking noise (positive Schroder-phase noise is used to calculate the SNR). TDT yielded the largest R^2^ values and lowest *p*-Values and showed the strongest associations to OCL-lmn, ONB-lmn, and MoCA. One possible reason for these findings is the global cognitive nature of both the TDT and -lmn/MoCA measures. The OCL and ONB-lmn measures are the mean of the log_10_ transformed reaction times for correct responses to tasks related to playing cards. The -lmn measures could be interpreted as combination of accuracy, speed, and memory. This is similar to the findings of Humes et al. (2013) and others, who showed the importance of global sensory-processing performance to global cognitive function (Humes et al., 2013; Humes, 2015; Deal et al., 2016; Glick and Sharma, 2020). The working memory components of the ONB and OCL tasks could also be related to auditory working memory, which has been shown to be an important component of language comprehension, even in the absence of background noise (Daneman and Merikle, 1996; Wingfield and Tun, 2007). The addition of background noise in the TDT may reduce auditory working memory capacity, resulting in the decreased ability to rehearse and recall a target, further compromising the perception of the signal already degraded by noise (Pichora-Fuller et al., 1995; Parbery-Clark et al., 2011). Age effects were also observed on ONB-lmn and OCL-lmn measures. The degree of age-related function on working memory has shown to be dependent of task difficult with larger age effects with higher cognitive demands (Salthouse and Meinz, 1995; Nilsson, 2003; Danielsson et al., 2019). The association of TDT to MoCA also provides evidence for a global hypothesis. The MoCA is a popular screening test for cognitive impairment that covers key cognitive domains including episodic memory, language, attention, orientation, visuospatial ability and executive functions (Nasreddine et al., 2005). In those with mild cognitive impairment speech-in-noise processing may increase the recruitment of neural networks involved in memory, attention, and learning to compensate for this dysfunction (Iliadou et al., 2017; Jalaei et al., 2019). Another hypothesized reason for the association between TDT and cognitive measures is the cross-cultural simplicity. While the Gap does not require any linguistic interpretation and the HINT sentences have been translated accurately into Swahili, the TDT provides a simple closed-set speech-in-noise task with minimal cultural or educational influences.

A surprising finding was that although both the HINT and TDT are speech-in-noise tasks, the TDT showed much stronger relationships with cognitive test results. Also, the unique pattern of the HINT relating to accuracy, and TDT relating to speed of working memory and visual learning on the ONB and OCL tasks was unexpected. Both the HINT and TDT are speech-in-noise tasks that use a SNR metric as the outcome variable. But the HINT is a composite score of three spatial orientations of the speaker and background noise. The HINT also uses speech-shaped noise to the long-term average of the presented sentences. Also, the HINT requires repeating back entire sentences, where the overall meaning of the sentence can provide some context to help the subject repeat it correctly. The TDT, however, requires remembering three unrelated numbers. The reasons for the differences between the tests are not clear, but does show that not all measures including speech in noise are the same.

Overall, our findings support the study of central auditory tests for measuring function on neurocognitive domains of learning and working memory regardless of HIV status. In the current study, we did not find strong evidence for HIV affecting the association between central auditory performance and cognitive measures. We hypothesized that PLWH would show differences in auditory processing due the diffuse effect of HIV on the central nervous system such as gray matter atrophy, diffuse white matter abnormalities in the internal capsule, thalamus, and corpus collosum, axonal injury, and loss of axonal density (Kuhn et al., 2018). We did find overall mean differences between HIV groups (Table 3), and differences in distribution of the scatter points for HIV– vs. HIV + individuals (Figure 1), but no significant difference in the relationship between central auditory and cognitive measures when age was in the model. These findings could be because many PLWH are performing to similar levels of those without the disease due to modern antiretroviral therapy. Additionally, it may be that only a subset of those with HIV show a difference in the relationship of central auditory tests and cognitive measures and this is not apparent in the overall measures. The CD4 levels of our cohort are consistent with HIV + individuals with well-controlled HIV on cART indicating that HIV was mostly well-controlled in this group. Also, it may be that although there were more PLWH with cognitive difficulties, the relationship between cognition and the central auditory variables is not different from what occurs due to aging. Our results support that age is a significant factor in the auditory-cognitive relationship. PLWH may be experiencing “accelerated aging” and may just be at a higher point on the curve relating cognition to central auditory test performance. Longitudinal analysis of this relationship is warranted to better assess this “accelerated aging” hypothesis. Regardless, if deficits in auditory function precede cognitive decline and ultimately brain health, early detection of cognitive deficits may be possible by evaluating aspects of central auditory processing.

We found central auditory tests were significantly associated with learning and working memory on the Cogstate, but not response time on the TOVA. The results are interesting because, except for providing instructions in performing the tests, none of the cognitive tests used in the study have an auditory component for their execution. By examining temporal acuity in an auditory gap detection paradigm, we found that age was significantly related to TOVA response time and Ex-Gaussian Mu with older adults having poorer response time compared to young adults. These observed age effects are consistent with previous studies that have suggested that age-related differences in complex measures of auditory temporal processing may be explained, in part, by age-related deficits in processing speed and attention (Snell, 1997; Humes et al., 2009, 2012; Harris et al., 2010; Humes, 2021). Age has a large effect on cognitive speed, which declines earlier and at a higher rate than memory (Salthouse, 2009). Humes et al. (2009) found a significant correlation between auditory and visual gap detection that was associated with general cognitive function, but not with processing speed on the Wechsler Adult Intelligence Scale (WAIS-III) in older adults (60-88 years). While there was a difference in age between cohorts and we used the TOVA instead of the WAIS-III, our results support previous findings that show gap detection is not related to processing speed when age is included in the model. Continuing to study gap detection and processing speed is important because they have been linked to age related changes in speech recognition, especially in acoustically complex conditions (Snell, 1997; Palmer and Musiek, 2014). But with the TOVA used in this study, we cannot endorse a significant relationship between processing speed and gap detection accounting for age in our experimental cohort.

The central auditory relationships to cognition are particularly important because central auditory tests can be easy to administer and do not require extensive training. Measures of central auditory processing are easy to train people on how to use, not complicated to administer, and not restricted to English. This makes them particularly appealing for use in resource-limited settings, such as developing nations. In contrast, cognitive tests can be challenging to train people how to use, are resource-intensive (e.g., materials), are often sensitive to education, and have typically been created for Western cultures. These are all barriers to their use in resource-limited, international settings. Most central auditory tests can be completed faster than traditional full cognitive test batteries (NIH toolbox takes approximately 2 h to complete) and some take less time than a cognitive screening test (10 min for the MoCA). The entire central auditory test battery (GAP, HINT, and TDT) used in this study took approximately 15 min, while the TOVA alone took over 20 min. These factors provide compelling arguments for exploring the use of central auditory tests to track cognitive performance in resource limited settings.

This study has some limitations. Previous work has demonstrated that age is an independent predictor of cognitive performance and speech in noise ability in HIV + adults (Zhan et al., 2017b). Processing speed is one of the strongest predictors of performance across cognitive tasks in adults (Salthouse and Ferrer-Caja, 2003) and age-related changes in processing speed are well established (Verhaeghen and Salthouse, 1997) and supported in this study. While age-related changes were not the focus of the current study, their influence on the auditory/cognitive relationship cannot be ignored. We observed an age effect on both TOVA measures, OCL-lmn, and ONB-lmn. Interestingly, all of these measures have a speed of processing component. Yet, even with age effects included in the model, central auditory tests were still significantly associated with cognitive function on the Cogstate.

Regarding gap detection testing and cognitive speed of processing, we only used gap detection thresholds as our primary outcome variable. There may be additional data within the test that were not fully utilized in this study. For example, measuring response time at the gap detection thresholds or even plotting a curve to each individual’s gap detection test from 0% correct at short gaps to 100% at longer gaps. Further development of these methods may produce a more sensitive test in detecting cognitive decline in HIV or other population with neurocognitive decline. Another limitation is that the present study does not address whether central auditory tests can be used to predict or track cognitive function in individuals over time. Examining the relationship between cognition and central auditory measures and whether this affected by HIV requires a longitudinal study. Instead, this study was focused on what cognitive domains were most strongly related to central auditory tests in this mixed cohort of normal hearing individuals that spanned a large age range. Result from this study do not prove causal relations, but only significant associations between central auditory and cognitive measures. Also, our test battery was limited in its scope. With a different set of cognitive tests (and additional domains), relationships might have been more robust. In addition, exploring the relationship of central auditory processing and cognition within HIV-infected individuals should be completed in populations outside of sub-Saharan Africa. This will allow for a better understanding of the generalizability of our findings. Further analysis of longitudinal CD4 count history may also provide a continuous metric of infection severity, which may be more predictive of cognitive dysfunction. With modern cART, lower CD4 counts have been exceedingly rare in our cohort and the general health of all subjects is consistent with well-managed HIV infection. Further longitudinal analysis of those who develop lower CD4 counts (e.g., <200 cell/μl) warranted. Examination of central auditory processing over time in HIV + individuals is also needed, as such work might provide further insight into the concept of accelerated aging in the brain in HIV-infected persons, and whether this responds to interventions.

## Conclusion

The overall results from the study suggest central auditory focused tests are positively related to cognitive function in our cohort, particularly in the areas of learning and working memory. The Gap test was not related to any cognitive measure with age in the model, while the HINT and TDT were related to learning and working memory. TDT scores were also found to be significantly related to the MoCA. We did not find any evidence of a HIV effect on the association of central auditory and cognitive measures. With longitudinal confirmation, central auditory tests, specifically speech-in-noise tests such as the TDT could provide an easy-to-use, quick method for assessing and potentially predicting cognitive dysfunction in those with HIV and other related cognitive deficits.

## Data Availability Statement

The raw data supporting the conclusions of this article will be made available by the authors, without undue reservation.

## Ethics Statement

The studies involving human participants were reviewed and approved by the Dartmouth College. The patients/participants provided their written informed consent to participate in this study.

## Author Contributions

CN completed the final data and statistical analyses, created the tables and figures, and completed the final draft of the manuscript. JL participated in the analysis plan, statistical analyses, interpretation of findings, and completed the initial complete draft of the manuscript. AM supervised the assessment team, coordinated the participant scheduling and follow-up, provided quality assurance, reviewed the data, and supported institutional review board approvals. JA completed preliminary data analyses, tables, and co-authored the completed first draft of the manuscript. AF verified data integrity, trained the assessment team, help to lead quality assurance for testing and data management, coordinated standard operating procedures, coordinated monthly conference call meetings for the study team, and supported institutional review board approvals. MH assisted in data analysis. CR verified data integrity and assisted with data interpretation. EM assisted with study management, and data review. MB participated in study design, test selection, and consulted to the project. NM assisted with study design and was primarily responsible for all aspects of study oversight in Tanzania. JB was study primary investigator, and participated in all phases of study conceptualization, study design, proposal writing, analysis plan, study implementation of protocols, interpretation, and manuscript editing. All authors contributed to the article and approved the submitted version.

## Conflict of Interest

The authors declare that the research was conducted in the absence of any commercial or financial relationships that could be construed as a potential conflict of interest.

## Publisher’s Note

All claims expressed in this article are solely those of the authors and do not necessarily represent those of their affiliated organizations, or those of the publisher, the editors and the reviewers. Any product that may be evaluated in this article, or claim that may be made by its manufacturer, is not guaranteed or endorsed by the publisher.

## Footnotes

1
www.Cogstate.com

## References

[B1] Anderson S. Kraus N. (2010). Sensory-Cognitive Interaction in the Neural Encoding of Speech in Noise: A Review. *J. Am. Acad. Audiol.* 21 575–585. 10.3766/jaaa.21.9.3 21241645 PMC3075209

[B2] Anderson S. White-Schwoch T. Parbery-Clark A. Kraus N. (2013). A dynamic auditory-cognitive system supports speech-in-noise perception in older adults. *Hear. Res.* 300 18–32. 10.1016/j.heares.2013.03.006 23541911 PMC3658829

[B3] Antinori A. Arendt G. Becker J. T. Brew B. J. Byrd D. A. Cherner M. (2007). Updated research nosology for HIV-associated neurocognitive disorders. *Neurology* 69 1789–1799. 10.1212/01.WNL.0000287431.88658.8b 17914061 PMC4472366

[B4] Bangirana P. Sikorskii A. Giordani B. Nakasujja N. Boivin M. J. (2015). Validation of the CogState battery for rapid neurocognitive assessment in Ugandan school age children. *Child Adolesc. Psychiatry Ment. Health* 9:38. 10.1186/s13034-015-0063-6 26279675 PMC4536703

[B5] Bloch M. Kamminga J. Jayewardene A. Bailey M. Carberry A. Vincent T. (2016). A Screening Strategy for HIV-Associated Neurocognitive Disorders That Accurately Identifies Patients Requiring Neurological Review. *Clin. Infect. Dis.* 63 687–693. 10.1093/cid/ciw399 27325690 PMC4981762

[B6] Boivin M. J. Chernoff M. Fairlie L. Laughton B. Zimmer B. Joyce C. (2019). African Multi-Site 2-Year Neuropsychological Study of School-Age Children Perinatally Infected, Exposed, and Unexposed to Human Immunodeficiency Virus. *Clin. Infect. Dis.* 71 e105–e114. 10.1093/cid/ciz1088 31848582 PMC7755090

[B7] Boivin M. J. Nakasujja N. Sikorskii A. Opoka R. O. Giordani B. (2016). A Randomized Controlled Trial to Evaluate if Computerized Cognitive Rehabilitation Improves Neurocognition in Ugandan Children with HIV. *Aids Res. Hum. Retrovir.* 32 743–755. 10.1089/aid.2016.0026 27045714 PMC4971428

[B8] Buckey J. C. Fellows A. M. Magohe A. Maro I. Gui J. Clavier O. (2019). Hearing complaints in HIV infection originate in the brain not the ear. *AIDS* 33 1449–1454. 10.1097/QAD.0000000000002229 30932961 PMC6602823

[B9] Centers for Disease Control and Prevention (2020). *Global HIV & Tuberculosis.* Available Online at: https://www.cdc.gov/globalhivtb/ [Accessed February 20, 2020 2020]

[B10] Craik F. I. (2007). The role of cognition in age-related hearing loss. *J. Am. Acad. Audiol.* 18 539–547. 10.3766/jaaa.18.7.2 18236642

[B11] Cysique L. A. Maruff P. Darby D. Brew B. J. (2006). The assessment of cognitive function in advanced HIV-1 infection and AIDS dementia complex using a new computerised cognitive test battery. *Arch. Clin. Neuropsychol.* 21 185–194. 10.1016/j.acn.2005.07.011 16343841

[B12] Daneman M. Merikle P. M. (1996). Working memory and language comprehension: a meta-analysis. *Psychon. Bull. Rev.* 3 422–433. 10.3758/BF03214546 24213976

[B13] Danielsson H. Humes L. E. Rönnberg J. (2019). Different Associations between Auditory Function and Cognition Depending on Type of Auditory Function and Type of Cognition. *Ear. Hear.* 40 1210–1219. 10.1097/AUD.0000000000000700 30807540 PMC6706331

[B14] Deal J. A. Betz J. Yaffe K. Harris T. Purchase-Helzner E. Satterfield S. (2016). Hearing Impairment and Incident Dementia and Cognitive Decline in Older Adults: the Health ABC Study. *J. Gerontol. Ser. A Biol. Sci. Med. Sci.* 72:glw069. 10.1093/gerona/glw069 27071780 PMC5964742

[B15] Du Plessis L. Paul R. H. Hoare J. Stein D. J. Taylor P. A. Meintjes E. M. (2017). Resting-state functional magnetic resonance imaging in clade C HIV: within-group association with neurocognitive function. *J. Neurovirol.* 23 875–885. 10.1007/s13365-017-0581-5 28971331 PMC5780332

[B16] Ettenhofer M. L. Foley J. Castellon S. A. Hinkin C. H. (2010). Reciprocal prediction of medication adherence and neurocognition in HIV/AIDS. *Neurology* 74 1217–1222. 10.1212/WNL.0b013e3181d8c1ca 20220123 PMC2865732

[B17] Gates G. A. Cobb J. L. Linn R. T. Rees T. Wolf P. A. D’agostino R. B. (1996). Central Auditory Dysfunction, Cognitive Dysfunction, and Dementia in Older People. *Arch. Otolaryngol. Head Neck Surg.* 122 161–167. 10.1001/archotol.1996.01890140047010 8630210

[B18] Glick H. A. Sharma A. (2020). Cortical Neuroplasticity and Cognitive Function in Early-Stage, Mild-Moderate Hearing Loss: evidence of Neurocognitive Benefit From Hearing Aid Use. *Front. Neurosci.* 14:93. 10.3389/fnins.2020.00093 32132893 PMC7040174

[B19] Grigoryan G. Fellows A. M. Musiek F. Chambers R. Clavier O. H. Buckey J. C. (2013). “Mathematical modeling of gap detection” in *Association for Research in Otolaryngology Midwinter Meeting.* (Baltimore: Association for Research in Otolaryngology).

[B20] Hallgren M. Larsby B. Lyxell B. Arlinger S. (2001). Cognitive effects in dichotic speech testing in elderly persons. *Ear Hear.* 22 120–129. 10.1097/00003446-200104000-00005 11324841

[B21] Hammers D. Spurgeon E. Ryan K. Persad C. Barbas N. Heidebrink J. (2012). Validity of a Brief Computerized Cognitive Screening Test in Dementia. *J. Geriatr. Psychiatry Neurol.* 25 89–99. 10.1177/0891988712447894 22689701

[B22] Hammers D. Spurgeon E. Ryan K. Persad C. Heidebrink J. Barbas N. (2011). Reliability of repeated cognitive assessment of dementia using a brief computerized battery. *Am. J. Alzheimer. Dis. Other. Demen.* 26 326–333. 10.1177/1533317511411907 21636581 PMC7469666

[B23] Harris K. C. Eckert M. A. Ahlstrom J. B. Dubno J. R. (2010). Age-related differences in gap detection: effects of task difficulty and cognitive ability. *Hear. Res.* 264 21–29. 10.1016/j.heares.2009.09.017 19800958 PMC2868108

[B24] Harris K. C. Wilson S. Eckert M. A. Dubno J. R. (2012). Human Evoked Cortical Activity to Silent Gaps in Noise. *Ear Hear.* 33 330–339. 10.1097/AUD.0b013e31823fb585 22374321 PMC3340542

[B25] Heaton R. K. Franklin D. R. Jr. Deutsch R. Letendre S. Ellis R. J. Casaletto K. (2015). Neurocognitive change in the era of HIV combination antiretroviral therapy: the longitudinal CHARTER study. *Clin. Infect. Dis.* 60 473–480. 10.1093/cid/ciu862 25362201 PMC4303775

[B26] Hoover E. C. Souza P. E. Gallun F. J. (2017). Auditory and Cognitive Factors Associated with Speech-in-Noise Complaints following Mild Traumatic Brain Injury. *J. Am. Acad. Audiol.* 28 325–339. 10.3766/jaaa.16051 28418327 PMC5600820

[B27] Humes L. E. (2015). Age-Related Changes in Cognitive and Sensory Processing: focus on Middle-Aged Adults. *Am. J. Audiol.* 24 94–97. 10.1044/2015_AJA-14-0063 25768926 PMC4610267

[B28] Humes L. E. (2020). Associations Between Measures of Auditory Function and Brief Assessments of Cognition. *Am. J. Audiol.* 29 825–837. 10.1044/2020_AJA-20-00077 32976027 PMC8608158

[B29] Humes L. E. (2021). Longitudinal Changes in Auditory and Cognitive Function in Middle-Aged and Older Adults. *J. Speech Lang. Hear. Res.* 64 230–249. 10.1044/2020_JSLHR-20-00274 33400551 PMC8608226

[B30] Humes L. E. Busey T. A. Craig J. C. Kewley-Port D. (2009). The effects of age on sensory thresholds and temporal gap detection in hearing, vision, and touch. *Atten. Percept. Psychophys.* 71 860–871. 10.3758/APP.71.4.860 19429964 PMC2826883

[B31] Humes L. E. Dubno J. R. Gordon-Salant S. Lister J. J. Cacace A. T. Cruickshanks K. J. (2012). Central Presbycusis: a Review and Evaluation of the Evidence. *J. Am. Acad. Audiol.* 23 635–666. 10.3766/jaaa.23.8.5 22967738 PMC5898229

[B32] Humes L. E. Kidd G. R. Lentz J. J. (2013). Auditory and cognitive factors underlying individual differences in aided speech-understanding among older adults. *Front. Syst. Neurosci.* 7:55. 10.3389/fnsys.2013.00055 24098273 PMC3787592

[B33] Husain F. T. Fromm S. J. Pursley R. H. Hosey L. A. Braun A. R. Horwitz B. (2006). Neural bases of categorization of simple speech and nonspeech sounds. *Hum. Brain Map.* 27 636–651. 10.1002/hbm.20207 16281285 PMC4770462

[B34] Idrizbegovic E. Hederstierna C. Dahlquist M. Kampfe Nordstrom C. Jelic V. Rosenhall U. (2011). Central auditory function in early Alzheimer’s disease and in mild cognitive impairment. *Age Ageing* 40 249–254. 10.1093/ageing/afq168 21233090

[B35] Iliadou V. V. Bamiou D.-E. Sidiras C. Moschopoulos N. P. Tsolaki M. Nimatoudis I. (2017). The Use of the Gaps-In-Noise Test as an Index of the Enhanced Left Temporal Cortical Thinning Associated with the Transition between Mild Cognitive Impairment and Alzheimer’s Disease. *J. Am. Acad. Audiol.* 28 463–471. 10.3766/jaaa.16075 28534735

[B36] Jalaei B. Valadbeigi A. Panahi R. Nahrani M. H. Arefi H. N. Zia M. (2019). Central Auditory Processing Tests as Diagnostic Tools for the Early Identification of Elderly Individuals with Mild Cognitive Impairment. *J. Audiol. Otol.* 23 83–88. 10.7874/jao.2018.00283 30727718 PMC6468277

[B37] Japee S. Holiday K. Satyshur M. D. Mukai I. Ungerleider L. G. (2015). A role of right middle frontal gyrus in reorienting of attention: a case study. *Front. Syst. Neurosci.* 9:23. 10.3389/fnsys.2015.00023 25784862 PMC4347607

[B38] Kamminga J. Bloch M. Vincent T. Carberry A. Brew B. J. Cysique L. A. (2017). Determining optimal impairment rating methodology for a new HIV-associated neurocognitive disorder screening procedure. *J. Clin. Exp. Neuropsychol.* 39 753–767. 10.1080/13803395.2016.1263282 28052738

[B39] Kotz S. A. Schwartze M. (2010). Cortical speech processing unplugged: a timely subcortico-cortical framework. *Trends Cogn. Sci.* 14 392–399. 10.1016/j.tics.2010.06.005 20655802

[B40] Koziol L. F. Budding D. E. (2009). *Subcortical Structures and Cognition : implications for Neuropsychological Assessment.* New York: Springer. 10.1007/978-0-387-84868-6

[B41] Kuhn T. Kaufmann T. Doan N. T. Westlye L. T. Jones J. Nunez R. A. (2018). An augmented aging process in brain white matter in HIV. *Hum. Brain Mapp.* 39 2532–2540. 10.1002/hbm.24019 29488278 PMC5951745

[B42] Maro I. Moshi N. Clavier O. H. Mackenzie T. A. Kline-Schoder R. J. Wilbur J. C. (2014). Auditory impairments in HIV-infected individuals in Tanzania. *Ear Hear.* 35 306–317. 10.1097/01.aud.0000439101.07257.ed 24441742 PMC3999286

[B43] Meinke D. K. Norris J. A. Flynn B. P. Clavier O. H. (2017). Going wireless and booth-less for hearing testing in industry. *Int. J. Audiol.* 56 41–51. 10.1080/14992027.2016.1261189 27976975 PMC5581305

[B44] Moore D. R. Edmondson-Jones M. Dawes P. Fortnum H. Mccormack A. Pierzycki R. H. (2014). Relation between Speech-in-Noise Threshold, Hearing Loss and Cognition from 40–69 Years of Age. *PLoS One* 9:e107720. 10.1371/journal.pone.0107720 25229622 PMC4168235

[B45] Nasreddine Z. S. Phillips N. A. BéDirian V. R. Charbonneau S. Whitehead V. Collin I. (2005). The Montreal Cognitive Assessment, MoCA: a Brief Screening Tool For Mild Cognitive Impairment. *J. Am. Geriatr. Soc.* 53 695–699. 10.1111/j.1532-5415.2005.53221.x 15817019

[B46] Niemczak C. Fellows A. Lichtenstein J. White-Schwoch T. Magohe A. Gui J. (2021). Central Auditory Tests to Track Cognitive Function in People With HIV: longitudinal Cohort Study. *JMIR Form Res.* 5:e26406. 10.2196/26406 33470933 PMC7902183

[B47] Nilsson L.-G. (2003). Memory function in normal aging. *Acta Neurol. Scand.* 107 7–13. 10.1034/j.1600-0404.107.s179.5.x 12603244

[B48] Overton E. T. Kauwe J. S. Paul R. Tashima K. Tate D. F. Patel P. (2011). Performances on the CogState and standard neuropsychological batteries among HIV patients without dementia. *AIDS Behav.* 15 1902–1909. 10.1007/s10461-011-0033-9 21877204 PMC3594991

[B49] Palmer S. B. Musiek F. E. (2014). Electrophysiological gap detection thresholds: effects of age and comparison with a behavioral measure. *J. Am. Acad. Audiol.* 25 999–1007. 10.3766/jaaa.25.10.8 25514452

[B50] Panza F. Quaranta N. Logroscino G. (2018). Sensory Changes and the Hearing Loss–Cognition Link. *JAMA Otolaryngol. Head Neck Surg.* 144:127. 10.1001/jamaoto.2017.2514 29222539

[B51] Parbery-Clark A. Strait D. L. Anderson S. Hittner E. Kraus N. (2011). Musical Experience and the Aging Auditory System: implications for Cognitive Abilities and Hearing Speech in Noise. *PLoS One* 6:e18082. 10.1371/journal.pone.0018082 21589653 PMC3092743

[B52] Pichora-Fuller M. K. Kramer S. E. Eckert M. A. Edwards B. Hornsby B. W. Humes L. E. (2016). Hearing Impairment and Cognitive Energy: the Framework for Understanding Effortful Listening (FUEL). *Ear Hear.* 37 5S–27S. 10.1097/AUD.0000000000000312 27355771

[B53] Pichora-Fuller M. K. Schneider B. A. Daneman M. (1995). How young and old adults listen to and remember speech in noise. *J. Acoust. Soc. Am.* 97 593–608. 10.1121/1.412282 7860836

[B54] Robertson K. Liner J. Heaton R. (2009). Neuropsychological assessment of HIV-infected populations in international settings. *Neuropsychol. Rev.* 19 232–249. 10.1007/s11065-009-9096-z 19455425 PMC2690834

[B55] Rönnberg J. Lunner T. Zekveld A. Sörqvist P. Danielsson H. Lyxell B. (2013). The Ease of Language Understanding (ELU) model: theoretical, empirical, and clinical advances. *Front. Syst. Neurosci.* 7:31. 10.3389/fnsys.2013.00031 23874273 PMC3710434

[B56] Rudner M. Lunner T. (2014). Cognitive spare capacity and speech communication: a narrative overview. *Biomed. Res Int.* 2014:869726. 10.1155/2014/869726 24971355 PMC4058272

[B57] Ruel T. D. Boivin M. J. Boal H. E. Bangirana P. Charlebois E. Havlir D. V. (2012). Neurocognitive and motor deficits in HIV-infected Ugandan children with high CD4 cell counts. *Clin. Infect. Dis.* 54 1001–1009. 10.1093/cid/cir1037 22308272 PMC3297647

[B58] Salthouse T. A. (2009). When does age-related cognitive decline begin? *Neurobiol. Aging* 30 507–514. 10.1016/j.neurobiolaging.2008.09.023 19231028 PMC2683339

[B59] Salthouse T. A. Ferrer-Caja E. (2003). What needs to be explained to account for age-related effects on multiple cognitive variables? *Psychol. Aging* 18 91–110. 10.1037/0882-7974.18.1.91 12641315

[B60] Salthouse T. A. Meinz E. J. (1995). Aging, inhibition, working memory, and speed. *J. Gerontol. B Psychol. Sci. Soc. Sci.* 50 297–306. 10.1093/geronb/50B.6.P297 7583809

[B61] Sardone R. Battista P. Panza F. Lozupone M. Griseta C. Castellana F. (2019). The Age-Related Central Auditory Processing Disorder: silent Impairment of the Cognitive Ear. *Front. Neurosci.* 13:619. 10.3389/fnins.2019.00619 31258467 PMC6587609

[B62] Saylor D. Dickens A. M. Sacktor N. Haughey N. Slusher B. Pletnikov M. (2016). HIV-associated neurocognitive disorder - pathogenesis and prospects for treatment. *Nat. Rev. Neurol.* 12:309. 10.1038/nrneurol.2016.27 27080521 PMC5842923

[B63] Snell K. B. (1997). Age-related changes in temporal gap detection. *J. Acoust. Soc. Am.* 101 2214–2220. 10.1121/1.418205 9104023

[B64] Song F. Zhan Y. Ford J. C. Cai D. C. Fellows A. M. Shan F. (2020). Increased Right Frontal Brain Activity During the Mandarin Hearing-in-Noise Test. *Front. Neurosci.* 14:614012. 10.3389/fnins.2020.614012 33390894 PMC7773781

[B65] Specht K. (2014). Neuronal basis of speech comprehension. *Hear. Res.* 307 121–135. 10.1016/j.heares.2013.09.011 24113115

[B66] Szymaszek A. Sereda M. Pöppel E. Szelag E. (2009). Individual differences in the perception of temporal order: the effect of age and cognition. *Cogn. Neuropsychol.* 26 135–147. 10.1080/02643290802504742 18988063

[B67] Verhaeghen P. Salthouse T. A. (1997). Meta-analyses of age-cognition relations in adulthood: estimates of linear and nonlinear age effects and structural models. *Psychol. Bull.* 122 231–249. 10.1037/0033-2909.122.3.231 9354147

[B68] Vissoci J. R. N. De Oliveira L. P. Gafaar T. Haglund M. M. Mvungi M. Mmbaga B. T. (2019). Cross-cultural adaptation and psychometric properties of the MMSE and MoCA questionnaires in Tanzanian Swahili for a traumatic brain injury population. *BMC Neurol.* 19:57. 10.1186/s12883-019-1283-9 30961532 PMC6454609

[B69] Watson B. U. (1991). Some Relationships between Intelligence and Auditory-Discrimination. *J. Speech Hear. Res.* 34 621–627. 10.1044/jshr.3403.621 2072686

[B70] White-Schwoch T. Magohe A. K. Fellows A. M. Rieke C. C. Vilarello B. Nicol T. (2020). Auditory neurophysiology reveals central nervous system dysfunction in HIV-infected individuals. *Clin. Neurophysiol.* 131 1827–1832. 10.1016/j.clinph.2020.04.165 32554244 PMC7363550

[B71] Wingfield A. Tun P. A. (2007). Cognitive supports and cognitive constraints on comprehension of spoken language. *J. Am. Acad. Audiol.* 18 548–558. 10.3766/jaaa.18.7.3 18236643

[B72] Wong P. C. M. Jin J. X. Gunasekera G. M. Abel R. Lee E. R. Dhar S. (2009). Aging and cortical mechanisms of speech perception in noise. *Neuropsychologia* 47 693–703. 10.1016/j.neuropsychologia.2008.11.032 19124032 PMC2649004

[B73] Zekveld A. A. Kramer S. E. Festen J. M. (2011). Cognitive load during speech perception in noise: the influence of age, hearing loss, and cognition on the pupil response. *Ear. Hear.* 32 498–510. 10.1097/AUD.0b013e31820512bb 21233711

[B74] Zhan Y. Fellows A. M. Qi T. Clavier O. H. Soli S. D. Shi X. (2017b). Speech in Noise Perception as a Marker of Cognitive Impairment in HIV Infection. *Ear Hear.* 39:1. 10.1097/AUD.0000000000000508 29112532 PMC5920702

[B75] Zhan Y. Buckey J. C. Fellows A. M. Shi Y. (2017a). Magnetic Resonance Imaging Evidence for Human Immunodeficiency Virus Effects on Central Auditory Processing: a Review. *J. Aids Clin. Res.* 8:708. 10.4172/2155-6113.1000708 28890843 PMC5589342

[B76] Zhan Y. Fellows A. M. Qi T. Clavier O. H. Soli S. D. Shi X. (2018). Speech in Noise Perception as a Marker of Cognitive Impairment in HIV Infection. *Ear. Hear.* 39 548–554. 29112532 10.1097/AUD.0000000000000508 PMC5920702

